# Role of teprotumumab in the treatment of active moderate-to-severe Graves’ orbitopathy

**DOI:** 10.1530/ETJ-22-0185

**Published:** 2022-12-08

**Authors:** Luigi Bartalena

**Affiliations:** 1 University of Insubria, Varese, Italy

Graves’ orbitopathy (GO), also named thyroid-associated orbitopathy (TAO) or thyroid eye disease (TED), is an autoimmune inflammatory disease of the orbit and the most important extrathyroidal manifestation of Graves’ disease (1). After an initial inflammatory (‘active’) phase of undefined duration, GO progressively burns out within 18–24 months but may cause fibrotic changes and remodeling of the orbital tissues, with abnormal appearance and visual dysfunction as sequelae (1). GO has a negative impact on the quality of life of affected individuals (2) because of its disfiguring (exophthalmos) and dysfunctional (diplopia) features and can only partially be prevented by intervening to change modifiable risk factors (3). In the large majority of cases, GO is mild, self-limiting, and nonprogressive (4), requiring only local measures (artificial tears, ophthalmic gels) and control of risk factors (5). Very rarely, GO is sight-threatening, due to compressive dysthyroid optic neuropathy (DON) and/or exophthalmos-related corneal breakdown, requiring prompt treatment with very high doses of i.v. glucocorticoids (ivGCs), local treatments, and/or orbital decompression surgery (5).

True challenges and therapeutic dilemmas are posed by active moderate-to-severe forms, representing <10% of cases (3). It is well established that any immunosuppressive/anti-inflammatory treatment should be carried out as early as possible, ideally within 6–9 months from the onset of disease, as this increases the chances of a favorable therapeutic outcome. High-dose GCs have been used for decades as the standard of care and are still a mainstay for the management of these forms, because of their potent anti-inflammatory effects and nonspecific immunosuppressive actions (6). They are highly effective for inflammatory symptoms and signs of GO, as well as for early onset diplopia, but their effect on exophthalmos appears to be limited (7, 8). Although GCs are efficacious when administered orally, ivGCs are more effective and better tolerated (1). Recent years have witnessed the development of new biologicals or their application to GO, aiming at specifically targeting relevant steps in the pathogenesis of GO (9). Some of these drugs, including rituximab (a monoclonal antibody depleting CD20-positive B cells) and tocilizumab (a monoclonal antibody directed against the interleukin-6 receptor), because of the limited available evidence, are currently not considered first-line treatments for active moderate-to-severe GO but may represent second-line therapies for GC-resistant forms (5). A major advance in this field has been provided by the use of teprotumumab, a monoclonal antibody inhibiting the insulin-like growth factor-1 receptor, which is involved, like the thyroid-stimulating hormone receptor, in the pathogenesis of GO (10). Two multicenter randomized clinical trials (RCTs) published in the *New England Journal of Medicine* in 2017 and 2020 showed that in patients with active moderate-to-severe GO of very recent onset (4–6 months), teprotumumab is highly effective for inflammatory changes, diplopia, and quality of life (11, 12). In addition, and at variance with other available therapies, teprotumumab was reported to be very effective for exophthalmos, with a marked reduction in exophthalmometer readings similar to that obtained after orbital decompression surgery (11, 12). A third RCT in which patients were treated/retreated with teprotumumab if they were either unresponsive to placebo, unresponsive to teprotumumab or flaring after teprotumumab, confirmed the short-term effectiveness of teprotumumab (13).

Last year, the European Group on Graves’ Orbitopathy (EUGOGO) Guidelines recommended ivGCs ± mycophenolate (an immunosuppressive agent with a dual anti-T and anti-B cells) as the first-line treatment for active moderate-to-severe GO and included teprotumumab among second-line treatments (5). In this issue of the *European Thyroid Journal*, a task force of experts from the American Thyroid Association (ATA) and the European Thyroid Association (ETA) publish a Consensus Statement on the management of thyroid eye disease; this format allowed them to avoid recommendations, while providing evidence-based suggestions/indications (key points) (14). While many key points are similar/superimposable to EUGOGO recommendations, a major difference is in regard to the role of teprotumumab. Key point 7.1.1.1 of the ATA/ETA Consensus Statement reads ‘Intravenous glucocorticoid (IVGC) is a preferred treatment for active moderate-to-severe TED when disease activity is the prominent feature in the absence of significant proptosis … or diplopia’. On the other hand, key point 7.1.3.1 reads ‘TEP [teprotumumab] is a preferred therapy, if available, in patients with active moderate-to-severe TED with significant proptosis … and/or diplopia’ (14). In spite of the cautious formulation used, the reader will understand that, if available, teprotumumab should be used as the first-line treatment in almost all cases, except, perhaps, for the mildest cases within the spectrum of active, moderate-to-severe GO. In fact, it is rare that significant inflammation (activity) is not associated with some degree of diplopia and/or exophthalmos, and *vice versa*.

I would like to argue that the presence of diplopia is not a contraindication to (or a reason to not prefer) ivGC treatment. In a review of 7 RCTs of ivGC totalling 149 patients, diplopia disappeared or improved in 60% of cases (7), quite a similar response to that observed after teprotumumab (15). In a large multicenter EUGOGO RCT, diplopia response was much lower (about 20%), but the mean duration of GO in the 3 arms of the study was long, from 10 to 18 months (vs 4–6 months in the 2 teprotumumab trials) (8). The clue from longstanding clinical practice and the available data is that diplopia of short duration, as with other manifestations of active GO, is more likely to be responsive to whatever immunosuppressive/targeted therapy (1). Accordingly, the only striking difference between ivGCs and teprotumumab, and a very important one indeed, resides with the higher effectiveness of the latter on exophthalmos, with a mean reduction of 2.46–3.32 mm in the 2 pivotal studies (11, 12) vs a mean reduction of 1.14 mm in 8 RCTs and 1.58 mm in 9 nonrandomized studies using ivGCs (7). A RCT comparing ivGCs and teprotumumab in exactly the same clinical setting is warranted to define the degree of this difference between the two therapies.

Relapse (or recrudescence of disease activity) is relatively common, around 30%, after ivGC withdrawal (8). Teprotumumab is not different in this regard. A pooled data analysis, subgroup analyses of 1 year off-treatment follow-up of the 2 pivotal studies showed a relapse of exophthalmos and diplopia in 33% and 31% of patients, respectively (15). Slightly fewer teprotumumab-treated patients (8) than placebo-treated patients (11) required additional medical/surgical treatments (15).

Safety is a major concern with any immunosuppressive/targeted therapy. Because high-dose GCs have been used for decades in thousands of patients, possible adverse of events of GCs in patients with GO are well known, including hepatotoxicity, risk of infections, hyperglycemia, osteoporosis, psychosis, depression, and death (7, 16). Accordingly, a careful selection of candidate patients and the exclusion of those with contraindications is mandatory (5). GC-related adverse events are very well described by both the EUGOGO Guidelines (5) and the ATA/ETA Consensus Statement (14). They are less frequent than in the past, because lower doses of ivGCs are currently used, and patients are carefully screened and followed in multidisciplinary specialized centers, set up in many European countries for more than 20 years thanks to EUGOGO. Teprotumumab is a novel drug, and the unknowns are currently more than the knowns with regard to safety (17). In the 2 pivotal trials using teprotumumab, ≥10% of patients had adverse events, usually graded as mild-to-moderate, which included muscle cramps, deterioration of blood glucose control, nausea, diarrhea, alopecia, and also 1 case of intracerebral hemorrhage (11, 12). Subsequently, one case of amyloid encephalopathy, responsive only to plasmapheresis, as well as a few cases of aggravation/*de novo* occurrence of inflammatory bowel disease have been described (18, 19). Very importantly, sensorineural hearing impairment has been reported in 15% of teprotumumab-treated patients, apparently persistent in 45% of them and not necessarily bound to preexisting otologic problems (20). Thus, in addition to a careful pretreatment screening and selection of patients, a faithful report of long-term adverse events is warranted to achieve sound information on the safety of this drug.

The cost of teprotumumab is also a major point. As reported by the ATA/ETA Consensus Statement, the cost of a course of teprotumumab is exceedingly high (and unaffordable by most healthcare systems), around several hundred thousand US dollars (depending on patient weight), about 2000-fold higher than a course of ivGCs (14). Teprotumumab has been approved in 2020 by the American Food and Drug Administration for the treatment of GO, with no limitation to the active moderate-to-severe forms investigated in the two pivotal studies, but it is currently not available worldwide and not approved in Europe. A recent survey among ATA/ETA members clearly reflected these geographical differences, as ivGCS were indicated as first-line treatment by around 70% of European specialists, while teprotumumab was preferred by a large proportion of American specialists (21). The same survey also underscored the limited availability and cost coverage of teprotumumab compared to high-dose GCs, either oral or iv. Availability does not necessarily mean that the drug is accessible if the costs of treatment are not covered. Cost and availability are two major issues to be solved, as accessibility to best care is a general ethical issue to avoid disparity of care.

Last but not least, teprotumumab has been shown to be an effective drug, as it is much better than placebo (11, 12). A comparative study with (iv)GCs, the standard of care for decades, is warranted and cannot be eluded, as well as a cost-effectiveness assessment.

To summarize the earlier discussion (Table 1), teprotumumab appears to be, at least in the short term, an effective drug for the management of active moderate-to-severe GO, with a striking effect on exophthalmos. Enthusiasm surrounding it is understandable. However, before discarding GCs as the first-line treatment, the following open and relevant issues regarding teprotumumab need to be addressed: (i) long-term durability of response; (ii) need for subsequent nonsurgical treatments and rehabilitative surgeries; (iii) evaluation of long-term safety; (iv) high cost; (iv) limited availability and accessibility; (v) lack of direct comparative studies with ivGCs in the same clinical setting; and (vi) lack of cost-effectiveness assessment.

**Table 1:** Management of active moderate-to-severe Graves’ orbitopathy by high-dose (oral or i.v.) glucocorticoids or teprotumumab.

	Glucocorticoids	Teprotumumab
Efficacy^a^	High	High
Relapse after treatment withdrawal	About 30%	About 30%
Need for subsequent treatments	Yes	Yes
Adverse events	Known	Long-term unknown
Availability	Broad	Limited^b^
Cost	Cheap	Very high
Comparative study with glucocorticoids	---	Not available

^a^Greater efficacy of teprotumumab on exophthalmos.

^b^Licensed only in USA, currently not available in Europe.

## Funding

The author declares no funding contributed to the writing of this article.

## Declaration of interest

The author has no conflict of interests to declare.
